# Effects of including a dog on treatment motivation and the therapeutic alliance in child and adolescent psychotherapy: study protocol for a randomized controlled trial

**DOI:** 10.1186/s13063-023-07854-4

**Published:** 2024-01-05

**Authors:** Wanda Arnskötter, Suzanne Martin, Susanne Walitza, Karin Hediger

**Affiliations:** 1 Faculty of Psychology, University of Basel, Basel, Switzerland; 2 Department of Child and Adolescent Psychiatry and Psychotherapy, University Hospital of Psychiatry Zurich, Zurich, Switzerland; 3 Department of Epidemiology and Public Health, Swiss Tropical and Public Health Institute, Allschwil, Switzerland; 4 Faculty of Psychology, Open University, Heerlen, Netherlands

**Keywords:** Motivation, Therapeutic alliance, Canine-assisted psychotherapy, Animal-assisted therapy, Children, Adolescents, Dog, Randomized controlled trial

## Abstract

**Background:**

Motivation and a therapeutic alliance are crucial for successful therapy. It is assumed that dogs can increase motivation and help support therapeutic relationships. This is one of the reasons for including dogs in psychotherapy. While the positive effects of psychotherapy with dogs have been documented over the past years, little is known about the underlying mechanisms of animal-assisted psychotherapy. This study therefore aims to investigate whether and how the presence of a dog affects motivation and the therapeutic alliance in child and adolescent psychotherapy.

**Methods:**

The study is a randomized controlled trial assessing motivation and the therapeutic alliance during the first five sessions of psychotherapy attended by children and adolescents with different psychiatric disorders. We will recruit 150 children and adolescents and randomly assign them to one of three conditions: (a) a dog is present but not integrated in the therapeutic narrative, (b) a dog is actively integrated in the therapeutic narrative, and (c) no dog is present. The children’s and adolescents’ evaluations of the therapeutic alliance and of their motivation will be assessed as the primary outcomes using standardized questionnaires before and after the first five therapy sessions as well as at follow-up. Further outcomes include the therapists’ evaluations of the therapeutic alliance and their motivation, treatment adherence of the children and adolescents, and treatment satisfaction of the children and adolescents, their parents, and of the therapists. Interventions are conducted by experienced therapists who regularly work with their dogs. Outcomes will be analyzed using general linear models, with the treatment group as a fixed factor and the baseline values as covariates.

**Discussion:**

This study provides information on the possible motivation and alliance-enhancing effects of integrating a dog into child and adolescent psychotherapy. This is relevant for practice, as these two components are strong predictors of therapy outcome. Moreover, the study will contribute to a better understanding of how a dog should be incorporated into psychotherapeutic settings. This can lead to a more purposeful inclusion of dogs in psychotherapy for children and adolescents.

**Trial registration:**

The trial was registered on ClinicalTrials.gov, NCT05384808, on 20 May 2022.

**Supplementary Information:**

The online version contains supplementary material available at 10.1186/s13063-023-07854-4.

## Administrative information

Note: the numbers in curly brackets in this protocol refer to item numbers from the SPIRIT checklist. The order of the items has been modified to group similar items (see http://www.equator-network.org/reporting-guidelines/spirit-2013-statement-defining-standard-protocol-items-for-clinical-trials/).

**Table:** 

Title {1}	Effects of including a dog on treatment motivation and the therapeutic alliance in child and adolescent psychotherapy: Study protocol for a randomized controlled trial. Trial acronym: CAP
Trial registration {2a and 2b}.	ClinicalTrials.gov ID: NCT05384808 Protocol ID: 2022–00304
Protocol version {3}	Version 1.4, 07.07.2023
Funding {4}	The study is funded by an Eccellenza grant awarded to Karin Hediger (PCEFP1_194591) by the Swiss National Science Foundation. Additional funding comes from the Department of Child and Adolescent Psychiatry and Psychotherapy, University Hospital of Psychiatry, University of Zurich.
Author details {5a}	Wanda Arnskötter, Faculty of Psychology, University of Basel, Basel, Switzerland; E-mail: wanda.arnskoetter@unibas.ch Suzanne Martin, Faculty of Psychology, University of Basel, Basel, Switzerland; Department of Child and Adolescent Psychiatry and Psychotherapy, University Hospital of Psychiatry Zurich, Zurich, Switzerland; E-mail: suzanne.martin@unibas.ch Susanne Walitza, Department of Child and Adolescent Psychiatry and Psychotherapy, University Hospital of Psychiatry Zurich, Zurich, Switzerland; E-mail: susanne.walitza@pukzh.ch Karin Hediger, Faculty of Psychology, University of Basel, Basel, Switzerland; Department of Epidemiology and Public Health, Swiss Tropical and Public Health Institute, Allschwil, Switzerland; Faculty of Psychology, Open University, Heerlen, Netherlands; E-mail: karin.hediger@unibas.ch
Name and contact information for the trial sponsor {5b}	Prof. Dr. Karin Hediger University of Basel Missionsstrasse 62 4055 Basel Switzerland Tel: + 41 61 207 65 80 E-mail: karin.hediger@unibas.ch
Role of sponsor {5c}	The study sponsor is the principal investigator. The funders do not have any role in the study design, in the collection, management, analysis, and interpretation of data, in writing the report, or in the decision to submit the report for publication.

## Introduction

### Background and rationale {6a}

Over the last two decades, psychotherapy research has increasingly focused on investigating the underlying mechanisms of the efficacy of psychotherapy to gain insights into what makes treatment successful. Research has shown that the therapeutic alliance has one of the strongest effects on therapeutic outcomes [1]. Motivation is also a crucial factor, especially at the beginning of psychotherapy. Many potential patients struggle with being motivated to seek therapy in the first place. Children and adolescents, who usually do not choose psychotherapy themselves, often do not exhibit a high motivation to commit to treatment [2]. It is therefore important for them to find access to psychotherapeutic support that is motivating and meaningful.

Animals have been shown to be highly motivating and to help build the therapeutic alliance in the context of animal-assisted therapy. They can activate implicit motives and intrinsic motivation in humans [3]. This implies that animals can enhance the intrinsic motivation for active participation in psychotherapy. For some people, it may be simpler to establish contact with a person in the presence of a dog; in such cases, the animal acts as a “social catalyst” [4]. Moreover, people are perceived as more trustworthy by others when they are accompanied by an animal [5]. The presence of an animal may thus positively affect the therapeutic alliance in psychotherapy.

Due to their popularity, long domestication history, and ability to accompany humans in different places, dogs are considered to be very suitable therapy animals and are among the most prominent species in animal-assisted therapies. Besides many reported beneficial effects of canine-assisted psychotherapy on symptomatology in different populations [6, 7], the presence of a dog and interaction with them have been shown to enhance patients’ motivation and the therapeutic alliance [3, 8, 9]. However, the outcomes are inconsistent, and studies have often been criticized for their insufficient design, small sample sizes, group settings, and lack of generalization.

Moreover, the underlying mechanisms of how animal-assisted interventions work are still unclear. While some studies have suggested that an animal’s mere presence can have stress-reducing effects in children [10], others have indicated that only physical contact with an animal leads to such effects [11–13]. In addition, the extent to which the animal was integrated into the therapeutic narrative has usually remained unclear and requires further investigation. In a study investigating canine-assisted psychotherapy in a group treatment for children who had experienced sexual abuse, the reduction of posttraumatic-stress symptoms, depression, and anxiety symptoms was greater when children heard therapeutic stories about the dogs that were present than when the dogs were present without stories [14]. Another study showed that the presence of a dog that was part of the therapeutic narrative led to a significant reduction of the pain experienced in an experimental setting, while the presence of a dog that was not part of the therapeutic narrative did not have such an effect [15]. These results indicate that the active inclusion of dogs in the therapeutic narrative can affect therapeutic mechanisms and outcomes.

To our knowledge, no study on animal-assisted therapy has investigated the therapeutic alliance and motivation in psychotherapy for children and adolescents as the primary outcome, even though these factors are known to be key elements for successful psychotherapy. In addition, there are no consistent data on how the way a dog is integrated into a psychotherapeutic setting affects outcomes. The aim of this study is therefore to investigate the effects of the presence of a dog on the therapeutic alliance and motivation at the start of psychotherapy for children and adolescents and to understand how a dog should be incorporated into a psychotherapeutic setting in order to be most effective.

### Objectives {7}

The general objective of this study is to evaluate the effects of the presence of a dog on motivation and the therapeutic alliance in psychotherapy for children and adolescents.

Our primary objectives are to investigate if the presence of a dog has effects on motivation and the therapeutic alliance and to study to what extent and how a dog should be integrated into psychotherapeutic interventions for children and adolescents, aged 9 to 17, with psychiatric disorders. Specifically, we want to investigate whether the dog needs to be part of the therapeutic narrative or whether the presence of a dog that is not part of the therapeutic narrative is also beneficial.

The secondary objectives are to investigate the therapists’ motivation and alliance with the child/adolescent, treatment adherence, and the treatment satisfaction of the children and adolescents, their parents, and the therapists.

### Trial design {8}

This study is designed as a three-armed randomized controlled trial with parallel groups. Each participant will be allocated to one of three conditions: (a) the presence of a dog that is not part of the therapeutic narrative, (b) the presence of a dog that is part of the therapeutic narrative, or (c) no dog present (control condition). Stratified block randomization with a permuted block size will be used to allocate participants to the three conditions. For each participating therapist, permuted blocks with block sizes of 3 or 6 children and adolescents will be generated and randomly allocated to the therapists.

## Methods: participants, interventions, and outcomes

### Study setting {9}

The interventions will take place in different psychiatric and psychotherapeutic facilities and private practices in Switzerland and the surrounding German-speaking countries. Therapists will be recruited to participate in the study by the study team. The participating therapists will look for possible study participants among their patients who want to start psychotherapy, ask about their interest in participating in the study, and provide the contact information of the study team. Patient recruitment will be conducted by the study team.

The therapists will conduct the therapy sessions according to their standard procedures without restrictions concerning therapeutic activities apart from instructions on how to integrate or not integrate a dog. The five therapy sessions will usually take place at intervals of one week. If an appointment is canceled, the treatment will be resumed 1 week later. In this case, the interval between appointments should not exceed 3 weeks (except for over long vacations). Appointment cancelations will be documented. The participating therapists will be experienced in conducting canine-assisted psychotherapy and will work regularly with their dogs. The dogs involved in the intervention will have been approved by the employer and checked by their veterinarian regularly. They will all be in good physical condition, used to working with children and adolescents, and will adhere to good basic obedience. The dogs will always have the opportunity to retreat from interactions with the patient if they wish to do so. Each therapist participating in the study will work with their own dog, so different dogs will be part of the study. All activities will be conducted in accordance with the guidelines of the International Association of Human-Animal Interaction Organizations (IAHAIO) to ensure human and animal welfare [16]. This study protocol follows the SPIRIT guidelines (Standard Protocol Items: Recommendations for Interventional Trials) [17].

### Eligibility criteria {10}

Inclusion and exclusion criteria:

Each therapist is required to meet the following criteria:

- Works with their own therapy dog;
- Has completed therapy training (accredited psychotherapist) or, if not completed, is far advanced in supervised therapy training;
- Works with children or adolescents aged 9 to 17 years.

Therapists who do not meet any of these criteria cannot participate in the study.

The children and adolescents are required to meet the following criteria:

- Is between 9 and 17 years of age;
- Is seeing the therapist for the first time;
- Has, along with their legal guardian, basic knowledge of either German or English so that they can fill out questionnaires;
- Receives informed consent from their legal guardian;
- Has a neutral or positive attitude toward dogs.

Children and adolescents who meet any of the following criteria cannot participate in the study:

- Exhibits acute psychosis or has early childhood autism;
- Is afraid of dogs;
- Exhibits an allergic reaction to dogs;
- Has behaved aggressively toward dogs;
- Has a diagnosed developmental disorder or intellectual delay or cannot complete the questionnaires due to developmental level.

### Who will take informed consent? {26a}

At the beginning of the study, the study team will e-mail therapists from different psychiatric or psychotherapeutic facilities and practices in Switzerland and the surrounding German-speaking countries to inquire about their interest and willingness to participate in the study. In addition, a flyer will be posted on different psychological networks with information for therapists to contact the study team if they are interested in participating in the study. Therapists will be given an information sheet about the content and a short description of the study procedure via email. If a therapist is interested in participating in the study, the study team will arrange a first meeting with the therapist to provide detailed information about the study procedure and check for the inclusion and exclusion criteria. All the participating therapists will provide signed informed consent. The therapists will then look for possible study participants among their patients aged 9 to 17 years, who want to start psychotherapy and will ask about their interest in participating in the study. They will provide the contact information of the study team so that the legal guardians of the patients can get in touch with the study team. If the families are interested in participating in the study, the study team will arrange an initial meeting with the family to inform them about the nature of the study, its purpose, the procedures involved, the expected duration, the potential risks and benefits, and any discomfort it may entail. Each participant will be informed that participation in the study is voluntary, that they may withdraw from the study at any time without giving reasons, and that withdrawing their consent will not affect their subsequent treatment. The study team will then check for the inclusion and exclusion criteria and ask the family canine-related questions (concerning their fear of dogs, allergic reactions to dogs, previous experience with dogs, and attitude toward dogs).

All the participants in the study will be provided with a participant information sheet and a consent form describing the study with sufficient information for them to make an informed decision regarding their participation in the study. They will have the possibility to ask questions, and they will be given at least one day between the first information and the request from the study team to decide whether to participate or not. If they have questions after the information conversation, they will be able to contact the study team via email or phone. Each participant’s legal guardian will provide written informed consent before the child or adolescent participates in any study procedure. The informed-consent process will be documented in the patient file, and any discrepancy with the process as described in this protocol will be explained. The participants will not receive any payment for their participation in the study. However, the therapists will receive a voucher from a pet shop for each patient they treat and collect data on within the study procedure. The children and adolescents will receive a cinema or zoo voucher as compensation for their participation.

### Additional consent provisions for collection and use of participant data and biological specimens {26b}

Not applicable. No additional data or biological specimens will be collected.

## Interventions

### Explanation for the choice of comparators {6b}

The control condition will consist of psychotherapeutic sessions in which no dog is present during the first five therapy sessions. Moreover, the dog will not be present until the follow-up assessment. This condition will serve as the comparator. Children and adolescents allocated to this condition will receive regular psychotherapy with no dog present. With this control condition, we want to compare how motivation and the therapeutic alliance are affected if a dog is present that is or is not integrated into the therapeutic narrative. The children and adolescents in the control condition will be able to choose if they want to meet the therapist’s dog and receive therapy with the dog after completing the follow-up and termination of data collection.

### Intervention description {11a}

Once the patients and their legal guardians agree to participate in the study, they will be invited to participate in the initial meeting with the therapist. No dog will be present during the initial meeting. During the initial meeting, a psychopathological report is taken by the individual therapists in order to ascertain the current symptomatology. Based on this, a suspected diagnosis is made on the basis of the International Classification of Disease (ICD). At the end of the initial meeting, the participants will be asked to complete two questionnaires as a baseline assessment. The legal guardian fills out the demographic data questionnaire. The therapists will also complete two questionnaires as baseline measurements at the end of the initial meeting with the family.

After the baseline assessment, the participants will be randomly allocated to one of the three conditions. The therapists will be informed about the allocation of their patients to plan their therapy schedule with or without their dog. The participants will not know the condition to which they are allocated until the first therapy session.

The two intervention conditions will consist of five 50-min sessions of canine-assisted psychotherapy, meaning that a dog will be integrated into a standard psychotherapeutic setting for children/adolescents. Additionally, the dog will be integrated into the therapy until the follow-up assessment has been completed. The control condition will consist of five 50-min sessions of regular psychotherapy with no dog being included. The dog will not be integrated into the therapy until the follow-up assessment has been completed. In one intervention condition, the dog will be present but not integrated into the therapeutic narrative, while in the other, the dog will be integrated into the therapeutic narrative. A mandatory therapeutic method will not be prescribed, but all the therapists either will be fully trained and accredited as psychotherapists or, if they are still in training, will be closely supervised. The therapy sessions will be structured in accordance with the individual therapeutic goals of each patient.

Overview of the three conditions:

1. Therapy sessions with a psychotherapist and the mere presence of a dog that is not integrated into the therapeutic narrative. The dog will be present in the therapy room and can interact with the child/adolescent, but it will not be specifically involved in the therapeutic process until the follow-up questionnaires are completed. The children and adolescents will be told that the dog is present because it would otherwise be alone at home.
2. Therapy sessions with a psychotherapist and a dog that is part of the therapeutic narrative and included in the therapeutic process until the follow-up questionnaires are completed. In this condition, the children and adolescents will be told that the dog is present because it is a therapy dog and that it is there to support the child or adolescent in difficult situations. The dog will thus be part of the therapeutic context. The psychotherapists will receive a manual with ideas for how to actively involve the dog in the sessions.
3. Therapy sessions with a psychotherapist but without a dog. A dog will not be present in the therapy room until the follow-up questionnaires are completed.

### Criteria for discontinuing or modifying allocated interventions {11b}

The intervention will be modified or ended if any unpredictable or adverse events occur. This could include if the child or adolescent exhibits an allergic reaction, fear, or aggressive behavior toward the dog. Aggressive behavior by the dog will also qualify as an adverse event. Adverse events will lead to exclusion from the study. The participants will be able to request to discontinue the study at any time without giving reasons. The therapists will be able to offer the participants psychotherapy outside the study, as they would have otherwise planned.

### Strategies to improve adherence to interventions {11c}

The therapists will be given a manual with instructions for the conditions with a dog to standardize the interventions as much as possible. The instructions will specify how the dog should be integrated into therapy and how to give a cover story for the dog’s presence in the condition where it is not actively integrated into the therapy. Adherence to the manual will be controlled using a self-reporting checklist. The children’s and adolescents’ adherence to the treatment will be monitored by the therapists and recorded as an outcome.

### Relevant concomitant care permitted or prohibited during the trial {11d}

Concomitant care or interventions will not be controlled, and no restrictions will be in place.

### Provisions for posttrial care {30}

No study-related posttrial care will be provided. However, the therapists may continue the started psychotherapy. If any harm occurs during the trial, participation in the study will end immediately. Any harm that occurs during the course of participation will be covered by trial insurance.

### Outcomes {12}

Primary outcomes:

- The children’s or adolescents’ perception of the therapeutic alliance will be operationalized by the change from baseline to post-measurement measured using the Fragebogen zur therapeutischen Beziehung für Kinder und Jugendliche (FTB-KJ; original English version: Therapeutic Alliance Scales for Children) [18, 19].
- The motivation of the children and adolescents will be operationalized by the change from baseline to post-measurement measured using the Situational Motivation Scale for Children (SMS-15) [20].

Secondary outcomes:

- The children’s or adolescents’ perception of the therapeutic alliance will be operationalized by the change from baseline to follow-up measured using the FTB-KJ [18].
- The motivation of the children and adolescents will be operationalized by the change from baseline to follow-up measured using the SMS-15 [20].
- The treatment satisfaction of the children and adolescents at post-measurement will be measured using the Fragebogen zur Beurteilung der Behandlung (FBB-P; Treatment-satisfaction questionnaire) [21].
- The treatment satisfaction of the children and adolescents at follow-up will be measured using the FBB-P [21].
- Treatment adherence will be assessed based on the therapists’ documentation of the attended and missed sessions as well as the time of dropout.

Further tertiary outcomes:

- The therapists’ perception of the therapeutic alliance will be operationalized by the change from baseline to post-measurement as measured using the Fragebogen zur therapeutischen Beziehung für Kinder und Jugendliche – Version für Therapeuten (FTB-T; original English version: Therapeutic Alliance Scales for Children—therapist version) [18, 19].
- The motivation of the therapists will be operationalized by the change from baseline to post-measurement assessed using a self-designed questionnaire with specific questions on motivation.
- The treatment satisfaction of the therapists will be assessed at post-measurement using the Fragebogen zur Beurteilung der Behandlung (FBB-T; Treatment-satisfaction questionnaire) for therapists [21].
- The treatment satisfaction of the legal guardians will be assessed at post-measurement and follow-up using the Fragebogen zur Beurteilung der Behandlung (FBB-E; Treatment-satisfaction questionnaire) for parents [21].

### Further assessed variables

Attitude toward dogs, previous experiences with dogs, psychiatric diagnosis, previous therapy experience, and demographic variables (age and gender) will also be assessed.

### Participant timeline {13}

**Fig. 1:**
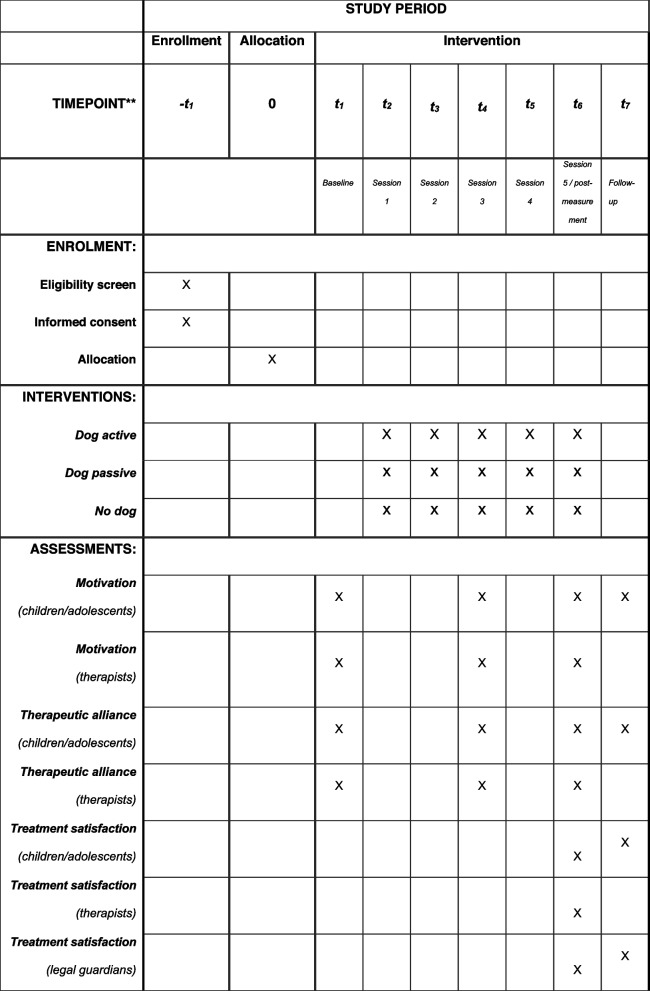
Participant timeline: time schedule for enrollment, intervention, and assessments during study period

The participant timeline is shown in Fig. 1. The assessments using questionnaires will take place before the first therapy session (baseline), after the third therapy session, after the fifth therapy session (post-measurement), and 4 weeks later (follow-up).

### Sample size {14}

Based on the results published by Klebanoff, Rosenau, and Wood [22] and Stefanini et al. [23], we assume a coefficient of variation of 0.2 and a true difference between the groups of at least 10%. We estimate that a total of 95 participants in the dog-present and dog-active groups combined and 48 participants in the control group are required to detect a difference in the primary analysis with a power of 80% at a 95% significance level. In order to have a balanced sample, we rounded up the anticipated total sample size to 150.

In the determination of the sample size ($n$_1_) for this study, the following formula for difference in means was employed:

$${n}_{1}=\frac{(r+1)}{r} \cdot \frac{{\sigma }^{2}{\left({Z}_{power}+ {Z}_{a/2}\right)}^{2}}{{{\text{difference}}}^{2}}$$

where:

$n$_1_ = size of smaller group.

$r$ = ratio of larger group to smaller group.

$\sigma$ = standard deviation of the characteristic.

difference = clinically meaningful difference in means of the outcome.

${Z}_{power}$ = corresponds to power (0.84 = 80% power).

${Z}_{a/2}$ = corresponds to two-tailed significance level (1.96 for a = 0.05).

Participants who complete less than three therapy sessions will remain in the intention-to-treat population, but additional participants will be recruited until the anticipated sample size of 150 for the available case population is achieved.

### Recruitment {15}

Therapists will be recruited through different therapeutic networks. A flyer will be published in different networks and magazines in the field of animal-assisted interventions to draw attention to the study. Children and adolescents will be directly recruited from the therapeutic facilities that the participating therapists work at and will be provided with contact information of the study team by the therapists.

## Assignment of interventions: allocation

### Sequence generation {16a}

Stratified block randomization with permuted block sizes will be used for allocating participants to the three conditions. For each participating therapist, permuted blocks with block sizes of 3 or 6 children and adolescents will be generated and randomly allocated to the therapists. The randomization lists will be computer-generated using a randomization program. The randomization lists will be password-protected documents that are only accessible to an independent person in charge of the randomization process.

### Concealment mechanism {16b}

The randomization lists will be generated by an independent scientific assistant. The lists will be password-protected documents that are only accessible to the independent scientific assistant. The assistant will communicate the allocations to the investigator. The investigator will inform the therapists of the allocated conditions.

### Implementation {16c}

The allocation sequences will be generated by a study-independent scientific assistant. After a participant has been enrolled in the trial by the investigator, the scientific assistant will assign the participant to a condition.

## Assignment of interventions: blinding

### Who will be blinded {17a}

The participants can only be blinded regarding the condition up until the first therapy session when they know whether the dog is present or not. However, study-related hypotheses will not be shared with the participants.

The therapists will be blinded until after the initial meeting with the patient and their family. Afterward, they will need to know if they have to plan the dog for the sessions to plan their schedules with the patients.

### Procedure for unblinding if needed {17b}

The design is open label, so unblinding will not occur.

## Data collection and management

### Plans for assessment and collection of outcomes {18a}

The principal investigator will be responsible for properly training all the involved study team members. Every team member will have access to written instructions and procedures (the standard operational procedures defined in the monitoring plan). Regular meetings for discussing the procedures will ensure that new information is shared with everyone and that there is a communication system for sharing new information. For quality assurance, the sponsor, the ethics committee (EKNZ), or an independent trial monitor may visit the research sites. Direct access to the source data and all study-related files will be granted on these occasions. All the involved parties will keep the participant data strictly confidential.

A second person of the study team will check a random sample of the data.

Online data collection via Lime Survey takes place before the first therapy session (baseline), after the third therapy session, after the fifth therapy session (post-measurement), and 4 weeks later (follow-up) (for an overview see study timeline, Fig. 1). Only the demographic data will be assessed using a paper–pencil questionnaire.

Participating patients and therapists are asked to fill out their online questionnaires at the end of the sessions. The outcomes of the first five patients and the first two therapists were assessed using paper–pencil questionnaires due to technical difficulties with the online questionnaires. From patient 6 onwards, only the online questionnaires were used to assure confidentiality in the participants’ answers and to reduce missing data. All participating therapists are asked to provide their patients with the necessary space and privacy while completing the online questionnaires.

#### Initial meeting (baseline)

Before the first therapy session, the participants will be invited for an initial meeting with their therapist. The aim of this contact will be to get to know each other and to assess baseline measurements for initial motivation and the therapeutic alliance. After the baseline assessment, the therapists will be informed which condition the patient is allocated to so that they can plan the next session.

#### End of the third and the fifth therapy sessions

The patients will fill out the questionnaires assessing motivation and the therapeutic alliance. The therapists will fill out their questionnaire assessing motivation and the therapeutic alliance. At the end of the intervention phase after the fifth session, the patients, their legal guardian, and the therapists will fill out the questionnaire assessing treatment satisfaction.

#### Follow-up

**Fig. 2:**
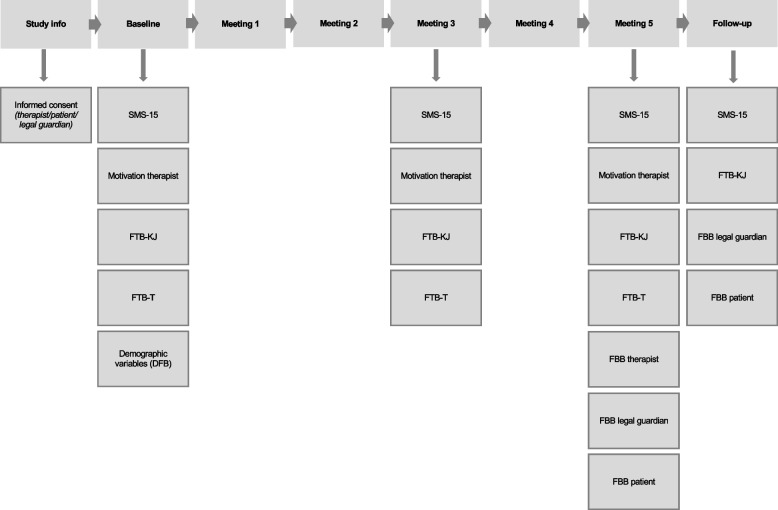
Study procedure. *SMS-15*, Situational Motivation Scale for Children; *FTB-KJ*, Fragebogen zur therapeutischen Beziehung für Kinder und Jugendliche (original English version: Therapeutic Alliance Scales for Children); *FTB-T*, Fragebogen zur therapeutischen Beziehung für Therapeuten (original English version: Therapeutic Alliance Scales for Children—therapist’s version); *FBB*, Fragebogen zur Beurteilung der Behandlung (Treatment-satisfaction questionnaire)

Four weeks after the first five sessions, the patients will be asked by their therapist to fill out the three questionnaires one more time. The legal guardians will also be asked to fill out the questionnaire on treatment satisfaction again (Fig. 2).

The following outcomes will be assessed: The children’s or adolescents’ perception of the therapeutic alliance as well as the therapists’ perception of the therapeutic alliance will be assessed using the FTB [18]. The questionnaire will be used at the initial meeting for baseline measurement, after the third therapy session, after the fifth therapy sessions (post-measurement), and 4 weeks later (follow-up). The questionnaire is a self-rating form with a total of 12 questions with a scale ranging from 1 (not at all) to 4 (very much). The FTB-KJ is validated for children and adolescents aged 6 to 18 years. Cronbach’s alpha is 0.82 for the total FTB-KJ (child and adolescent version) score and 0.83 for the total FTB-T score (therapist version). Retest reliability coefficients for the therapist version range from 0.61 to 0.79, whereas the coefficients for the child version are in some cases considerably lower for individual scales (0.10 to 0.64).

The motivation of the children and adolescents will be assessed using the SMS-15 [20]. The questionnaire will be used at the initial meeting for baseline measurement, after the third therapy session, after the fifth therapy session (post-measurement), and 4 weeks later (follow-up). The questionnaire is a self-rating form with a total of 15 questions related to the main question “Why are you currently receiving therapeutic treatment?” with a scale ranging from 1 (I definitely disagree) to 7 (I definitely agree). For the younger participants, we replaced the numbers with emojis for better readability. We will additionally ask a specific question about the dog in the two conditions with a dog: “How do you like having a dog in therapy?”, with a scale ranging from 1 (I don’t like it at all) to 7 (I like it very much). The SMS-15 is validated for children and adolescents aged 11 to 15 years. Cronbach’s alpha coefficient is 0.84 for the subscales of introjected motivation and regulation by identification and 0.82 for the subscales of intrinsic motivation, external regulation, and amotivation. Retest reliability for the total SMS-15 scale is *r* = 0.73, with *r* = 0.79 for the intrinsic motivation subscale, *r* = 0.76 for the external regulation subscale, *r* = 0.73 for the regulation by identification subscale, *r* = 0.71 for the introjected regulation subscale, and *r* = 0.67 for the amotivation subscale.

The motivation of the therapists will be assessed using a self-designed questionnaire with specific questions on motivation. The questionnaire will be used at the initial meeting for baseline measurement, after the third therapy session, and after the fifth therapy sessions (post-measurement). The questionnaire is a self-rating form with a total of six questions on a scale ranging from 1 (not at all true) to 4 (exactly true).

Treatment satisfaction at post-measurement and at follow-up will be measured using the FBB [21] for therapists (FBB-T), patients (FBB-P), and legal guardians (FBB-E). The questionnaire will be used after the fifth therapy sessions (post-measurement) as well as 4 weeks later (follow-up). The questionnaire is a self-rating form. The questionnaire for patients includes a total of 20 questions, the questionnaire for legal guardians contains 21 questions, and the questionnaire for therapists includes a total of 18 questions. All questions are answered on a scale from 0 (not at all) to 4 (exactly). The internal consistency for the different questionnaire versions with their subscales was tested to be on average with Cronbach’s alpha coefficients higher than 0.80. Retest reliability for all three questionnaire versions ranges between 0.68 and 0.77.

Treatment adherence will be assessed by documenting the attended and missed sessions as well as time of dropout.

### Plans to promote participant retention and complete follow-up {18b}

Participation in the study will be canceled if a child/adolescent or their legal guardian wishes to do so. Other criteria for withdrawal include if the child/adolescent experiences negative effects from the participation with the dog or if the dog is harmed by the child/adolescent. A voucher will serve as compensation for the study completion, and the study team will provide as much support as possible for all the participants to make data collection easy and to increase complete follow-up. All dropouts will be recorded, and all available data will be analyzed in the analyses. However, if study participants withdraw, more children/adolescents will be recruited until the planned number of participants with a complete dataset is reached.

### Data management {19}

The data will be recorded in an electronic case report form (eCRF) on Excel spreadsheets. An audit trail will be maintained by saving the Excel files under a new file name each time the data is transferred. Moreover, all changes made to the data will be indicated by adding a comment (indicating the name of the person adding changes and the date of the change). An eCRF will be maintained for each participant. The questionnaire data will be collected electronically via LimeSurvey or on paper. The electronic data will then be downloaded and copied directly into Excel files. Paper–pencil records will directly be transferred to the Excel files. Only the investigators and involved students will be authorized to enter the data into the eCRFs. All members of the study team will undergo training regarding data management and study-related procedures as defined in the standard operating procedures of the monitoring plan. No double data entry will be performed, but data entry will be controlled by a study team member through random checks, and range checks for the data values will be performed before data analysis.

Each participant receives an identification number when being enrolled in the study. Each participant will have an identification form with biographic data, such as birth date and the names of relatives. This sheet will be stored password protected and separately from all the other data together with the signed informed consent.

The source data will be available on site to document the existence of the study participants and will only be included in study-specific records. The source data will consist of the informed consent forms, the original paper questionnaires, and the electronically collected data via LimeSurvey.

The physical data provided on paper will be stored at the University of Basel in a secured archive. All electronic data will be stored on a university server that is password-protected. Only the investigator and the study team members have access to the password. There will be regular safety backups of all the data on the server to ensure that the data will remain accessible in case of unintended or unauthorized changes or deletions.

All the study data will be archived for 10 years after the termination or premature termination of the study. The data will be destroyed after the statutory period of recordkeeping. The final anonymized data will be made publicly available at the end of the study.

### Confidentiality {27}

The trial and participant data will be handled with uttermost discretion and will only be accessible to authorized personnel who require the data to fulfill their duties within the scope of the study. All the data will be pseudoanonymized, meaning that participants will only be identified by a unique participant number on the eCRFs and other study-specific documents. Published data will be fully anonymized.

This study is conducted in compliance with the protocol, the current version of the Declaration of Helsinki [24], the ICH-GCP [25], and the HRA [26] as well as other locally relevant legal and regulatory requirements. Moreover, the investigators follow the declarations and guidelines of the IAHAIO [16].

### Plans for collection, laboratory evaluation, and storage of biological specimens for genetic or molecular analysis in this trial/future use {33}

Not applicable because we are not using biological samples for genetic or molecular analysis in the study.

## Statistical methods

### Statistical methods for primary and secondary outcomes {20a}

#### Primary analyses

The participants’ motivation and perception of the therapeutic alliance at post-measurement will be analyzed using general linear models. The treatment group will serve as a fixed factor. The baseline values of the primary outcomes will serve as covariates. To assess the robustness of the model, an additional analysis adjusting for sex and age will be performed. We expect only a small proportion of missing data. The primary analysis will use the available case population analyzed according to the intention-to-treat principles.

#### Secondary analyses

Secondary outcomes will be analyzed in the same way as the primary analyses. Afterward, a per-protocol analysis will be done including only participants who complete and evaluate all five therapy sessions.

The statistical analyses will be conducted with R [27]. Descriptive analyses will be conducted. For categorical variables, the descriptive analyses will consist of raw numbers and percentages, for continuous variables, the mean, median, standard deviation, and range will be reported. For the primary analyses, we will report *p* values with a level of significance of 5% (*p* < 0.05). Results with a probability of error equal to or lower than 10% (*p* < 0.10) will be treated as indicating a trend. Model diagnostics will be performed visually and will include the normality of the residuals. Jan Hattendorf (Swiss TPH) will serve as the trial statistician. Any deviation from the original statistical plan will be described and justified in the final trial report and also reported in all publications.

### Interim analyses {21b}

Since the intervention does not include any medication, no exceptionally strong negative or positive effects are expected that would justify an interim analysis or formal stopping rules.

### Methods for additional analyses (e.g., subgroup analyses) {20b}

To assess the robustness of the model, an additional analysis adjusting for sex and age will be performed. Moreover, per-protocol analyses will be done including only participants who complete and evaluate all five therapy sessions.

### Methods in analysis to handle protocol nonadherence and any statistical methods to handle missing data {20c}

The data will be analyzed using the available case population according to the intention-to-treat principles. Missing data at the fifth visit will be replaced by the corresponding values after the third visit, if applicable. Experience shows that withdrawals are rare. In case the proportion of missing data exceeds 10%, we will use multiple imputation to analyze the intention-to-treat population.

### Plans to give access to the full protocol, participant level-data and statistical code {31c}

Anonymized participant-level data and the statistical code will be published open access via the Open Science Framework (OSF) platform.

## Oversight and monitoring

### Composition of the coordinating center and trial steering committee {5d}

The trial steering committee comprises three key members: one principal investigator and two co-investigators. This committee ensures the effective management of the trial. Duties encompass the approval of the final protocol and monitoring the ongoing progress of the study. The committee also has the authority to consider and agree upon modifications to the study protocol and the investigators’ brochure to facilitate a successful execution of the study. Meetings take place every 2 months.

The trial management committee, comprising the two co-investigators, master students, and one independent scientific assistant, is responsible for study planning and day-to-day management of the trial. The role of the group is to monitor all aspects of the conduct and progress of the trial, engage in the patient recruitment process, ensure that the protocol is adhered to, and take appropriate action to safeguard participants and the quality of the trial itself. The independent scientific assistant will be only responsible for generating the allocation sequences of the randomization. Meetings are held weekly.

Master’s students and interns will be included in the conduction of the study as part of their master’s projects or internships at the University of Basel.

### Composition of the data monitoring committee, its role and reporting structure {21a}

An internal trial monitor independent of the study team will be responsible for regular onsite monitoring according to the monitoring plan.

### Adverse event reporting and harms {22}

Therapists agree to report any adverse events occurring over the course of their participation in the study. Adverse events refer to their participation as well as to the participating children and adolescents. All adverse events will be collected, assessed, and reported, according to the Ordinance on Clinical Trials in Human Research (ClinO) [28]. Both investigator and sponsor-investigator make a causality assessment of the event to the trial intervention. Any event assessed as possibly, probably, or definitely related will be classified as related to the trial intervention. Each adverse event will be classified in its severity as mild, moderate, or severe. *Mild* means that the complication is tolerable, *moderate* that it interferes with daily activities, and *severe* that it renders daily activities impossible. Severe adverse events will be reported within 24 h to the sponsor-investigator and within 15 days to the ethics committee (EKNZ).

### Frequency and plans for auditing trial conduct {23}

The project management group meets every week to assess the progress and management of the ongoing trial. In parallel, the trial steering group assembles every 2 months throughout the trial’s duration to oversee its direction. Furthermore, an initial site initiation visit is carried out by an independent data monitoring entity before the enrollment of the first trial subject. Subsequently, the first monitoring visit is scheduled after half of the participants have been recruited, and a closure visit is planned upon completion of the recruitment process.

The Ethics Committee in Switzerland (EKNZ) does not engage in regular, pre-scheduled monitoring activities as this study was categorized as a low-risk intervention. However, the ethics committee or any other authority will be allowed to conduct an auditing trial at any time. If conducted, the process will be independent of the investigator and the sponsor. Additionally, any alterations or modifications made during the audit process must receive authorization from the ethics committee. An interim report is submitted to the ethics committee once annually to provide an overview of the trial’s progress and compliance with ethical guidelines.

### Plans for communicating important protocol amendments to relevant parties (e.g., trial participants, ethical committees) {25}

Any occurring changes to the study protocol will be sent immediately to the sponsor and funder. The principal investigator will inform the study center about the changes made. A copy of the revised protocol will be sent to the principal investigator to add to the Investigator Site File. Any deviation from the protocol will be fully documented using a breach report form. Substantial changes to the study setup, organization, protocol, or documents will be submitted to the ethics committee (EKNZ) for approval before implementation. Under emergency circumstances, deviations from the protocol to protect the rights, safety, and well-being of human subjects may proceed without prior approval of the EKNZ. Such deviations will be documented and reported to the EKNZ as soon as possible. Substantial amendments are changes that affect the safety, health, rights, or obligations of participants, changes in the protocol that affect the study objective(s) or central research topic, and changes of the study site(s) or of the study leader and sponsor (ClinO, Art. 29) [28].

A list of all nonsubstantial amendments will be submitted once a year to the EKNZ together with the annual safety report. The updated protocol will be uploaded to the clinical trial registry.

Any deviation from the original statistical plan will be described and justified in the final trial report and reported in all publications.

### Dissemination plans {31a}

The trial results will be communicated at internal meetings of the study team, will be presented at international congresses in the fields of psychology and animal-assisted interventions, and will be published in international peer-reviewed journals as open-access articles. Moreover, the results will be communicated to practitioners through informal publications or book chapters.

Participants who wish to be informed about the study results will be able to leave their address and will receive a summary of the results.

## Discussion

The aim of this study is to gain insights into the effects that integrating a dog into psychotherapy for children or adolescents has on motivation and the therapeutic alliance and into the extent to which a dog should be integrated into the therapeutic narrative in order to be beneficial.

The literature has often claimed that integrating a dog into psychotherapy enhances children’s motivation to attend sessions and facilitates the therapeutic alliance with the therapist. However, this has not been directly tested in a naturalistic setting. In contrast to previous research, motivation and the therapeutic alliance are the primary outcomes in this study, and we will measure both with standardized instruments at different time points. Moreover, we will assess motivation and the therapeutic alliance also from the therapists’ perspective, and we will ask the children/adolescents, therapists, and parents about treatment satisfaction.

To minimize confounding as much as possible, we will randomize the conditions and will not inform the therapists, children/adolescents, or parents about the condition they are allocated to until the first therapy session (after the baseline).

Since we will work with different practitioners, there will be variance in how the therapists work as well as in how they work with their dogs. As this can be a challenge for internal validity, we will provide the therapists with a manual that describes in detail how a therapy session in the study condition should be structured and which animal-assisted elements should be included. In addition, the manual will provide the therapists with a variety of materials as ideas for therapy. However, we do not want to interfere too much with real-life practice, and we want to conduct the study in a naturalistic setting to enhance its external validity.

Moreover, we will control for the effects of the individual therapists and type of therapy by assigning all conditions to each therapist using stratified block randomization. In the event that therapists opt for an early withdrawal from the study or encounter challenges in engaging children or adolescents willing to participate, achieving a balanced allocation to all three conditions becomes uncertain. Should such circumstances arise, we will transparently acknowledge this limitation and incorporate it into our result interpretation discussions.

We expect a broad range of different psychological disorders in the course of the study that might lead to variance in outcomes. However, since motivation and alliance are not necessarily dependent on psychological disorders, we do not expect the variance of data to be limiting for interpretation of our findings. Also, we intentionally chose not to make any distinction regarding the different disorder patterns in order to keep external validity high and to focus on the practice of canine-assisted therapy. Therefore, this study can give insight into how dogs affect motivation and the therapeutic alliance among a broad spectrum of psychological disorders.

We have noticed that recruiting therapists for this study is challenging because some of them always have their dogs with them in the practice and would have difficulties conducting the control condition. Moreover, we have noticed that a lot of families explicitly contacted the therapists for dog-assisted psychotherapy. For many patients, the prospect of being allocated to a condition where the dog is not present is a reason to decline study participation. Nevertheless, we aim to conduct this study as a randomized controlled trial to ensure good interpretability of the results.

One limitation of our study arises from the age range covered by the SMS-15 questionnaire, which is validated for individuals between the ages of 11 to 15 years. While our study involved children/adolescents aged 9 to 17, the questionnaire may not be equally valid across all age groups within our sample. Consequently, any comparisons between data from younger (9–10 years) and older (16–17 years) children/adolescents need to be approached carefully, as the questionnaire’s validity may vary outside the established age range.

With this study, we hope to provide insight into underlying mechanisms of animal-assisted psychotherapy and to add to the evidence basis of why animal-assisted psychotherapy can be effective. It is crucial not only to determine whether integrating a dog into psychotherapy has an effect but also to understand in what way this integration of an animal should be done. By comparing dogs that are part of a therapeutic narrative with dogs that are not part of a therapeutic narrative, we aim to increase the knowledge about how animals should best be integrated into a therapeutic setting in order to be most effective.

The knowledge gathered in this study will add to describing the mechanisms underlying animal-assisted therapy and help identify under what conditions the integration of a dog is beneficial.

## Trial status

The trial is currently running based on the latest study protocol version 1.4_07.07.2023. We submitted three amendments since the first decree of the original study protocol (Version 1.1_02.05.2022) due to minor changes to the study procedure.

Amendments included the following: developmental disorder or intellectual delay has been added to the exclusion criteria; vouchers were added as compensation for participants; the recruitment radius was extended to German-speaking countries surrounding Switzerland (Germany, Austria, Liechtenstein); randomization procedure was modified to stratified block randomization with permuted block sizes; an additional qualitative survey was conducted as part of a Master’s thesis, which has led to minor additions to the study procedure; an additional flyer was designed to be sent in the course of the recruitment phase.

Recruitment began in June 2022 and will approximately be completed in December 2024. By date of submission, 4.7% of all patients have been recruited. No drop-outs have been reported so far. Regarding the therapists, there is no maximal number to reach. By the date of submission, we have recruited 10 therapists, who participate in the study.

### Supplementary Information

**Additional file 1.** SPIRIT Checklist.

## Data Availability

The final trial dataset will be published open access.

## Abbreviations

ClinO: Ordinance on Clinical Trials in Human Research
eCRF: Electronic case report form
EKNZ: Ethikkommission Nordwest- und Zentralschweiz
FBB-E: Fragebogen zur Beurteilung der Behandlung (Treatment-satisfaction questionnaire) for legal guardians
FBB-P: Fragebogen zur Beurteilung der Behandlung (Treatment-satisfaction questionnaire) for patients
FBB-T: Fragebogen zur Beurteilung der Behandlung (Treatment-satisfaction questionnaire) for therapists
FTB-KJ: Fragebogen zur therapeutischen Beziehung für Kinder und Jugendliche (original English version: Therapeutic Alliance Scales for Children)
FTB-T: Fragebogen zur therapeutischen Beziehung für Therapeuten (original English version: Therapeutic Alliance Scales for Children—therapist’s version)
HRA: Human Research Act
IAHAIO: International Association of Human-Animal Interaction Organizations
ICH-GCP: International Council for Harmonisation of Technical Requirements for Pharmaceuticals for Human Use – Good Clinical Practice
OSF: Open Science Framework
SMS-15: Situational Motivation Scale for Children

## References

[1] Flückiger C, Del Re AC, Wampold BE, Horvath AO (2018). The alliance in adult psychotherapy: a meta-analytic synthesis. Psychotherapy (Chic).

[2] Marker CD, Comer JS, Abramova V, Kendall PC (2013). The reciprocal relationship between alliance and symptom improvement across the treatment of childhood anxiety. J Clin Child Adolesc Psychol.

[3] Wohlfarth R, Mutschler B, Beetz A, Kreuser F, Korsten-Reck U (2013). Dogs motivate obese children for physical activity: key elements of a motivational theory of animal-assisted interventions. Front Psychol.

[4] Corson SA, Corson EO’L, Gwynne PH, Arnold LE (1977). Pet dogs as nonverbal communication links in hospital psychiatry. Compr Psychiatry.

[5] Beetz A, Schöfmann I, Girgensohn R, Braas R, Ernst C (2019). Positive effects of a short-term dog-assisted intervention for soldiers with post-traumatic stress disorder—a pilot study. Front Vet Sci.

[6] Kamioka H, Okada S, Tsutani K, Park H, Okuizumi H, Handa S (2014). Effectiveness of animal-assisted therapy: a systematic review of randomized controlled trials. Complement Ther Med.

[7] Hediger K, Wagner J, Kunzi P, Haefeli A, Theis F, Grob C (2021). Effectiveness of animal-assisted interventions for children and adults with post-traumatic stress disorder symptoms: a systematic review and meta-analysis. Eur J Psychotraumatol.

[8] Collier T, Bennett P, Rohlf V, Howell T (2022). The effect of dog presence on the therapeutic alliance: a systematic review. Vet Sci.

[9] Jones MG, Rice SM, Cotton SM (2019). Incorporating animal-assisted therapy in mental health treatments for adolescents: A systematic review of canine assisted psychotherapy. PLoS One.

[10] Friedmann E, Katcher AH, Thomas SA, Lynch JJ, Messent PR (1983). Social interaction and blood pressure: influence of animal companions. J Nerv Ment.

[11] Binfet JT, Green FLL, Draper ZA (2021). The importance of client–canine contact in canine-assisted interventions: a randomized controlled trial. Anthrozoos.

[12] Beetz A, Kotrschal K, Turner DC, Hediger K, Uvnäs-Moberg K, Julius H (2011). The effect of a real dog, toy dog and friendly person on insecurely attached children during a stressful task: an exploratory study. Anthrozoos.

[13] Grossberg JM, Alf EF, Vormbrock JK (1988). Does pet dog presence reduce human cardiovascular responses to stress?. Anthrozoos.

[14] Dietz TJ, Davis D, Pennings J (2012). Evaluating animal-assisted therapy in group treatment for child sexual abuse. J Child Sex Abus.

[15] Wagner C, Gaab J, Hediger K (2023). The importance of the treatment rationale for pain in animal-assisted interventions: a randomized controlled trial in healthy participants. J Pain.

[16] International Association of Human-Animal Interaction. The IAHAIO definitions for animal assisted intervention and guidelines for wellness of animals involved in AAI. IAHAIO; 2014. Available from: http://pat.org.za/wp-content/uploads/2021/04/IAHAIO-WHITE-PAPER-TASK-FORCE-FINAL-REPORT_2018.pdf. updated 2018.

[17] Chan A-W, Tetzlaff JM, Gøtzsche PC, Altman DG, Mann H, Berlin J, Dickersin K, Hróbjartsson A, Schulz KF, Parulekar WR, Krleža-Jerić K, Laupacis A, Moher D (2013). SPIRIT 2013 Explanation and Elaboration: guidance for protocols of clinical trials. BMJ.

[18] Kronmüller KT, Hartmann M, Reck C, Victor D, Horn H, Winkelmann K (2003). Die therapeutische Beziehung in der Kinder- und Jugendlichen-Psychotherapie. Z Klin Psychol Psychother.

[19] Shirk SR, Saiz CC (1992). Clinical, empirical, and developmental perspectives on the therapeutic relationship in child psychotherapy. Dev Psychopathol.

[20] Skalski S. The Situational Motivation Scale (SMS-15) for Children: design and preliminary psychometric properties assessment. Konteksty Pedagogiczne. 2019;(1):191–203.

[21] Mattejat F, Remschmidt H (1998). Fragebögen zur Beurteilung der Behandlung (FBB).

[22] Klebanoff SM, Rosenau KA, Wood JJ (2019). The therapeutic alliance in cognitive-behavioral therapy for school-aged children with autism and clinical anxiety. Autism.

[23] Stefanini MC, Martino A, Allori P, Galeotti F, Tani F (2015). The use of animal-assisted therapy in adolescents with acute mental disorders: a randomized controlled study. Complement Ther Clin Pract.

[24] World Medical Association. World Medical Association Declaration of Helsinki: Ethical Principles for Medical Research Involving Human Subjects. JAMA. 2013;310(20):2191–4. 10.1001/jama.2013.281053. 10.1001/jama.2013.281053 24141714

[25] International Council for Harmonisation of Technical Requirements for Pharmaceuticals for Human Use – Good Clinical Practice (ICH-GCP). Available from: https://www.ema.europa.eu/en/ich-e6-r2-good-clinical-practice-scientific-guideline. Accessed 31 Aug 2023.

[26] Human Research Act (HRA). Available from: https://www.admin.ch/opc/de/classified-compilation/20061313/index.html.

[27] R Core Team. R: A Language and Environment for Statistical Computing. Vienna, Austria; 2016. Available from: https://www.R-project.org/

[28] Ordinance on Clinical Trials in Human Research. Available from: https://www.admin.ch/opc/de/classified-compilation/20121176/index.html. Accessed 29 Aug 2023.

